# Blood-Based Multi-Cancer Early Detection Tests (MCEDs) as a Potential Approach to Address Current Gaps in Cancer Screening

**DOI:** 10.1177/10732748241307360

**Published:** 2024-12-05

**Authors:** Chantelle Carbonell, John M. Hutchinson, Robert J. Hilsden, Huiming Yang, Darren R. Brenner

**Affiliations:** 1 Department of Oncology, 213572 University of Calgary, Calgary, AB, Canada; 2 Department of Medicine, Cumming School of Medicine, 213572 University of Calgary, Calgary, AB, Canada; 3 Screening Programs, 3146 Alberta Health Services, Calgary, AB, Canada

**Keywords:** multi-cancer early detection, review article, cancer screening, supplementary cancer testing, DNA testing, cancer management

## Abstract

Screening and early detection is one of the most effective approaches to reduce the population-level impact of cancer. Novel approaches to screening such as multi-cancer early detection tests (MCEDs) may further reduce cancer incidence and mortality. Many MCEDs detect fragments of circulating DNA containing mutations that originated from tumour cells, thereby informing both the presence of cancer and the cell-type of origin. In this review, we examine the current evidence of MCEDs as a potential tool to improve population-based cancer outcomes. We review the role of MCEDs to address low participation rates, disparities among underserved populations, changing epidemiology of common cancers, and the absence of screening tests for many cancer types. MCEDs have the potential to increase participation in cancer screening programs, as they may be less invasive than other procedures, and can screen for multiple cancer types in one appointment. Additionally, due to the lack of specialized collection equipment needed for these tests, underscreened populations and targeted populations could gain greater access to screening. Finally, because MCEDs can detect cancer types without screening tests that are moderately common and increasing in western populations, efficacious tests for these sites could alleviate the cancer burden and improve patient outcomes. While these tests offer great promise, considerable limitations and evidence gaps must be addressed. Notable limitations include scenarios where early detection does not improve survival outcomes, the costs and impact on health care resources for false positives, and false reassurance with subsequent lack of adherence to existing screening protocols.

## Introduction

Cancer is a leading cause of morbidity and death worldwide.^1^ In 2022, nearly 20 million new cancer cases and 9.7 million cancer-related deaths were reported worldwide and by 2050, the global cancer burden is estimated to increase to 35 million projected new cancer cases (a 77% increase from 2022).^1^ In Canada, cancer is the leading cause of death and it is estimated that approximately 1 in 4 Canadians will die from the disease.^2^ Further efforts and intervention are required to reduce the cancer burden.

Cancer screening with fully established tests is often associated with better patient outcomes, as cancer has a lesser impact when detected early.^2,3^ Cancer screening through population-based screening programs aims to detect pre-cancerous cells and/or cancers at an earlier stage when treatments are more likely to be effective, leading to improved patient and population outcomes.^2^ To date, effective screening tests and subsequent organized programs include only a few cancer types (breast, cervical, colorectal, lung, and prostate).^4-8^ Typically, single modality or target screening for disease works best when the disease incidence is high and effective treatments are available, hence why traditional screening programs have focused on these common cancers.

Advances in the era of precision medicine and genomic testing for disease has led to the development of multi-cancer early detection tests (MCEDs). MCEDs are a rapidly evolving branch of cancer detection and diagnosis technologies which can detect both the presence of cancer and the specific tissue of origin (TOO).^9-11^ The majority of MCEDs function by detecting and classifying cell-free tumour DNA (CF-DNA) in the blood.^10,12^ These DNA fragments can be released by cancerous or pre-cancerous cells and may contain specific mutations or methylation patterns which are involved in elements of cancer such as accelerated growth or immune evasion and can help to identify the tumour location and malignancy.^10^ MCEDs can often be performed with only a blood sample (primarily with blood plasma) and can identify types of cancer that do not currently have an established screening test.^13,14^ These cancer types comprise 60% of cancer-related deaths, making MCED clinically useful.^13^ Due to the simplicity of a blood draw, MCEDs can be less invasive for patients than other screening procedures and could lead to greater levels of detection. In these early-stages of research, MCEDs should primarily be used in conjunction with conventional screening techniques (colonoscopies, mammograms etc.).^12^ Recent studies have evaluated MCED performance, and the results of large, published studies are summarized in Table 1.

**Table 1.** Summary of Multi-Cancer Early Detection Blood Tests and their Published Screening Test Performance.

Test Name (Company Name, Country)	Study Name (Study ID)	Evaluation(s) Done	Study Type	Biological Signal	Age Range, years	Proportion of Women, %	Source of Cancers	No. of Cancer Types Detected	Specificity, % (95%CI)	Sensitivity, % (95%CI)	Tumor of Origin Accuracy, % (95%CI)	Positive Predictive Value, % (95%CI)	Negative Predictive Value, % (95%CI)
Galleri (GRAIL, USA)	Circulating cell-free Genome tlas (NCT02889978)^14,56-58^	Discovery, training and validation, clinical validation	Prospective, case-control multi-center, observational study	Cell-free DNA methylation	20+	55.4	Clinic	>50	99.5 (99.0-99.8)	51.5 (49.6-53.3)	88.7 (87.0-90.2)	44.4 (28.6-79.9)	99.4 (99.4-99.5)
	PATHFINDER (NCT04241796)^52,59^	Feasibility in outpatient settings for adults over age 50 without symptoms of cancer	Prospective, single-arm multi-center, observational study	Cell-free DNA methylation	50+	65.0	Clinic	36	99.1 (98.9-99.3)	NA	85 (60.9-93.6)	38.0 (28.8-48.3)	98.6 (98.3-98.9)
	SYMPLIFY (ISRCTN10226380)^51,60^	Efficacy in patients with symptoms that may be due to cancer	Prospective, case-control multi-center, observational study	Cell-free DNA methylation	18+	66.1	Clinic	24	98.4 (98.1-98.8)	66.3 (61.72-71.1)	85.2 (79.8-89.3)	75.5 (70.5-80.1)	97.6 (97.1-98.0)
	NHS-Galleri (ISRCTN91431511)^61,62^	Evaluate whether the MCED test can reduce the incidence rate of late-stage cancer in asymptomatic individuals	Pragmatic, prospective, simple (individual-level), randomised controlled trial	Cell-free DNA methylation	50-77	Trial ongoing, results pending	Population	Trial ongoing, results pending	Trial ongoing, results pending	Trial ongoing, results pending	Trial ongoing, results pending	Trial ongoing, results pending	Trial ongoing, results pending
SPOT-MAS (gene solutions, Vietnam)	K-DETEK (NCT05227261)^63-67^	Development Technical and clinical validation	Prospective, case-control multi-center, observational study	Circulating tumour DNA	40+	57.8	Clinic	5	99.8 (99.7-99.9)	78.1 (61.3-89.0)	84.0 (65.4-93.6)	58.1 (43.3-71.6)	99.9 (99.8-100.0)
MCDBT-1/2 (Shanghai Zhongshan Hospital, China)	THUNDER (NCT04820868)^68,69^	Development, training and validation Independent validation	Prospective, case-control single-blind multi-center, observational study	Cell-free DNA methylation	40-75	54.8	Clinic	6	MCDBT-1:98.9 (97.6-99.7)	MCDBT-169.1 (64.8-73.3)	MCDBT-183.2 (78.7-87.1)	NA	NA
									MCDBT-295.1 (92.8-96.9)	MCDBT-275.1 (71.9-79.8)	MCDBT-279.4 (74.9-83.5)		
OncoSeek (SeekIn, China)	A panel of seven protein tumour markers for effective and affordable multi-cancer early detection by artificial intelligence: a large-scale and multicentre case–control study^70^	Development, training and validation, Independent validation	Retrospective, case-control, multi-center, observational study	Cell-free DNA and protein tumour markers	Range not provided	46.4	Clinic	9+^a^ (all targeted)	92.9 (92.3-93.5)	51.7 (49.4-53.9)	66.8 (NA)	65.8 (63.3-68.1)	87.9 (87.2-88.6)
GuardantLUNAR-2 test (guardant health, USA)	Abstract 2141: Development of a highly-sensitive targeted cell-free DNA epigenomic assay for early-stage multi-cancer screening^71^	Development, feasibility on four cancer types with differing screening clinical utility	Prospective, case-control, multi-site observational study	Cell-free DNA methylation	18+	NA	Clinic	4^b^ (all targeted)	Classification threshold of 90-95	76-92 (NA)	98 (NA)	NA	NA
	SHIELD (NCT05117840)^72,73^	Clinical validation for cancer-risk cohorts	Prospective, observational, multi-site basket design trial without randomization	Cell-free DNA methylation	50-80	Trial ongoing, results pending	Clinic	Trial ongoing, results pending	Trial ongoing, results pending	Trial ongoing, results pending	Trial ongoing, results pending	Trial ongoing, results pending	Trial ongoing, results pending
	ECLIPSE (NCT04136002)^24,74^	Clinical validation for average risk colorectal cancer	Prospective, observational multi-site study without randomization	Cell-free DNA methylation	45-84	53.7	Clinic	1^c^ (targeted)	89.9 (89.0-90.7)	83.1 (72.2-90.3)	NA	NA	NA
CancerSEEK (exact Sciences corporation, USA)	ASCEND (NCT04213326)^75,76^	Development, technical and clinical validation	Prospective, case-control, observational study	Cell-free DNA circulating proteins and mutations	22+	51	Clinic	8^d^	>99 (NA)	62 (NA)	83 (NA)	NA	NA
	DETECT-A (NA)^77^	Feasibility and safety in women not previously known to have cancer	Exploratory prospective, interventional study	DNA and protein biomarkers	65-75	100	Population	9^e^ (all targeted)	98.9 (98.7-99.1)	27.1 (18.5-37.1)	NA	19.4 (13.1-27.1)	99.3 (99.1-99.4)
PanSeer (Singlera Genomics, USA)^78^	NA	Development, technical validation	Retrospective, longitudinal observational study	Cell-free DNA methylation	35-85	34	Population	5^f^	96.1 (92.5-98.3)	95.0 (89.0-98.0)	NA	NA	NA

cfDNA = cell-free DNA; MCED = multi-cancer early detection; NA = not available.

^a^Colorectal, lung, breast, liver, lymphoma, stomach, pancreatic, ovarian, oesophageal, other.

^b^Colorectal, lung, pancreas, bladder.

^c^Colorectal

^d^Ovarian, liver, stomach, pancreatic, esophageal, colorectal, lung, breast.

^e^Ovarian, lung, uterine, thyroid, colorectal, breast, lymphoma, kidney, unknown primary.

^f^Stomach, esophageal, colorectal, lung, liver.

**Figure 1.**
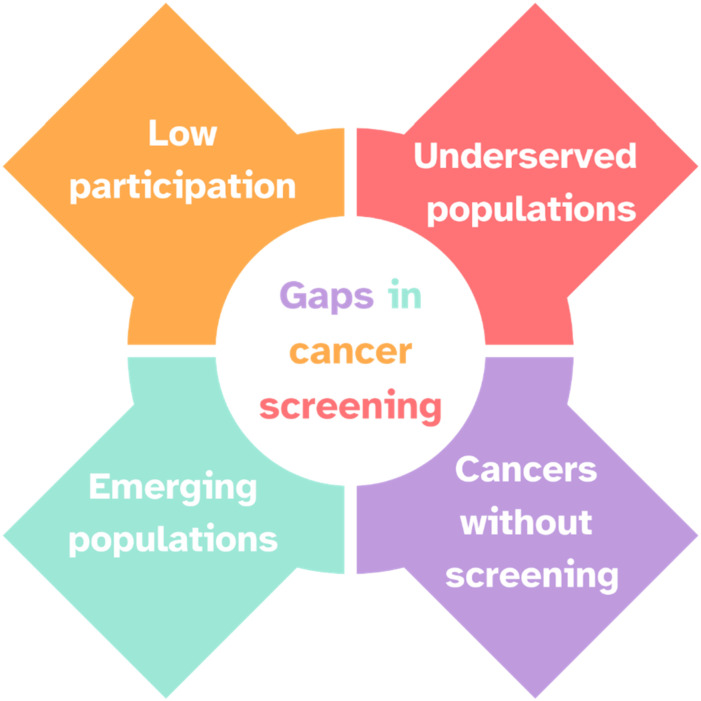
Gaps in cancer screening.

Population-based cancer screening has been a major driving force of reductions in cancer incidence and mortality. However, challenges and gaps to further impact have emerged. This paper will overview the current gaps in population-based cancer screening programs, including low participation rates, disparities among underserved populations, emerging trends in younger demographics, and the absence of screening tests for many cancer types (Figure 1). For each gap, we will highlight the potential of MCEDs to offer solutions to mitigate these challenges. The primary objective of this paper is to highlight the overall potential of MCEDs to reduce cancer burden and improve public health, while also discussing the existing evidence gaps for MCEDs and potential pitfalls.

## Low Screening Participation Rates

Screening participation is below recommended levels in many populations.^15^ A study of women in Ontario, Canada found that only 52.4% of eligible women were up-to-date for both breast cancer screening (mammograms) and cervical cancer screening (Pap test).^16^ This situation has been worsened by the COVID-19 pandemic, where many cancer screening programs across different sites were impacted and interrupted due to public health measures.^17-19^ This has also resulted in a backlog of procedures causing delays in currently assigned screening tests and diagnostic follow-up.^19^ While rates of screening showed recovery after the beginning of the pandemic, it is important to consider future interruptions and potential methods of mitigation.^19^

In the case of invasive screening procedures like colonoscopies, patients report fear of pain or feeling embarrassed being a major factor in keeping them from undergoing screening, which may be a cause of sub-optimal participation.^20,21^ A similar phenomenon can be seen with breast cancer screening, where studies found between 25–46% of women in England who did not reattend scheduled mammograms cited pain as the primary reason.^22^

MCEDs are less invasive and could alleviate many of the obstacles which prevent individuals from undergoing screening. Unlike many of the more invasive or time-consuming cancer screening tests like colonoscopies or mammograms, most MCEDs only require a single blood draw from the patient.^10^ While conventional screening should not be replaced by MCED screening, it could serve as a safety net to ensure a base level of screening in patients who refuse other forms of testing. A previous study surveyed the attitudes toward introduction of MCEDs of 1700 adults in the United States and found that the 74% of participants preferred tests involving blood draws (such as MCEDs) to more invasive procedures.^23^ Previous studies involving single-cancer early detection tests (SCEDs) have also shown high participation, such as the ECLIPSE study, which found that 89.6% of participants enrolled adhered to the blood-based tests.^24^

Due to the more convenient and time-efficient nature of MCEDs, they could potentially be included in regular physician check-ups, reducing the need for additional resource-intensive appointments. This may also make MCED testing less susceptible to the same interruptions which occurred during the COVID-19 pandemic. Additionally, procedures which cause pain or discomfort for patients could potentially be performed less frequently with the addition of MCEDs.^21,25^ Finally, because MCEDs are designed to detect many types of cancer, low participation across several cancer screening programs (breast, colorectal, cervical etc.) could be partially alleviated by higher participation in MCED screening programs, helping to reduce the overall cancer burden while maintaining cost-effectiveness.^13^

## Under Screened and Underserved Populations

Screening rates remain low among marginalized communities, including racial and ethnic minorities, low-income individuals, rural and remote populations, and those with limited access to healthcare.^26-30^ For example, 2022 United States data show that the age-adjusted prevalence among adults aged 50–75 years who reported being up-to-date with colorectal cancer screening was 74.6% (95% CI: 74.1–75.1) among white, non-Hispanic adults compared to 65.6% (95% CI: 61.8–69.1) among Asian and Pacific Islander, non-Hispanic adults, 61.7% (95% CI: 59.7–63.6) among Hispanic adults, and 61.1% (95% CI: 56.4–65.7) among American Indian and Alaska Native, non-Hispanic adults.^31^ Similar trends were reported for 2020 United States data on cervical cancer screening.^31^ Barriers to uptake of cancer screening in these populations include lack of awareness and support, discrimination in and mistrust of the health care system, cultural beliefs, language barriers, financial constraints, and inadequate healthcare infrastructure.^27,28,32,33^ Transportation challenges for rural patients, such as traveling long distances to see a clinician, have been associated with lower screening rates.^28^ With longer travel times, there is a need for available health care providers with open office hours during evenings and weekends; however, rural patients are less likely to access providers outside of regular business hours.^34^ Consequently, delays in diagnoses and timely intervention result in disproportionately higher cancer incidence, morbidity, and mortality rates.^35,36^ In Canada, screening participation rates are lower among First Nations, Inuit and Métis communities compared to non-Indigenous people.^37-39^ However, there are limited data available on First Nations, Inuit and Métis cancer research, underscoring the need for improved health record systems that include appropriately collected ethnocultural identifiers to better understand health trends in underserved minorities.^39^ First Nations, Inuit and Métis populations experience unique health inequities and barriers to healthcare access across all levels (individual, systems, structural).^40^ These barriers, such as those related to racism and discrimination, intergenerational trauma and abuse, and the lack of culturally competent care, decrease the likelihood for First Nations, Inuit and Métis people to participate in cancer screening programs.^40^ First Nations, Inuit and Métis groups living in rural and remote communities and those that are low-income face additional barriers to accessing healthcare and cancer screening programs.^40^

In contrast to conventional screening tests, MCEDs can be conducted in a centralized lab following a blood-draw from a health professional within the community, allowing cancer screening to be more convenient, time-efficient, and feasible for distribution to underserved populations.^32^ Moreover, MCEDs may reduce the frequency of appointments with primary care providers (PCPs) for cancer screening, which may help address the shortage of PCPs. In Alberta, Canada, the Enhanced Access to Cervical and Colorectal Cancer Screening (EACS) pilot project showed that adding cervical and colorectal cancer screening (Screen Test-EACS) to a current mobile breast cancer screening program (Screen Test) aimed towards reaching individuals in rural and remote communities increased overall screening rates for cervical (Screen Test-EACS: 27.5% vs Screen Test: 10.1%) and colorectal (Screen Test-EACS: 22.5% vs Screen Test: 10.9%) cancers by mitigating access barriers.^41^ If MCEDs are incorporated in existing mobile screening programs, these alternative modes of delivery could improve access to cancer screening services across multiple cancer types for individuals facing transportation barriers.

## Emerging Populations and Shifting Epidemiology

Over time, the epidemiology of many cancers are continually shifting due to changes in risk and exposures. For example, in recent years, younger people (less than 50 years old) have shown increasing cancer rates.^42,43^ A variety of cancer types, including colorectal cancer, and breast cancer have demonstrated increasing trends in younger groups.^13,43-45^ Based on a cohort study within the United States, there was an increase in early-onset cancers (cancers in people less than 50 years of age) from 99.96 cases per 100,000 individuals in 2010 to 102.97 cases per 100,000 individuals in 2019.^43^ This represents a major public health concern, and further public health measures must be taken to ensure these cases are detected and treated in early stages.

Screening for cancer types like colorectal and breast cancer commonly begin at an age of 50, as that has generally been determined to be when the risk becomes high enough to justify the balance of costs and benefits.^46^ Minimum screening ages have been lowered in some countries to respond to increasing rates, including Austria and Japan lowering their colorectal cancer screening age to 40 years old.^46^ Additionally, the United States Preventive Services Task Force (USPSTF) now recommends beginning breast cancer screening at 40 years old and colon cancer screening at 45 years old.^47,48^ These trends has also been seen within Canada, where the province of Alberta has lowered the minimum screening age for breast cancer to 45 years old.^49^

As the introduction of MCEDs would require revisions and expansions to existing screening programs, it represents an opportunity to modify screening recommendations for multiple age groups simultaneously. MCEDs could be administered to expanded age groups, in addition to what is currently offered with existing screening modalities. The increase in early-onset cancer is seen across several cancer types, meaning that multi-cancer tests are well suited to detect these new cases, rather than relying on individual cancer screening programs to each reduce the minimum recruitment age.^43^ Additionally, introduction of MCED screening as a single program for a wider age range could potentially allow for lesser costs compared to reducing the minimum screening age across multiple screening programs. As clinical knowledge of MCED tests expands, introduction into these emerging populations may become possible, however it is important to consider the early stage of this research.

## Cancers with No Existing Screening Tests

As previously mentioned, organized, population-based screening programs are only available for a few cancer types (breast, cervical, colorectal, lung, and prostate).^4-8^ Globally, these cancer types accounted for 44% of all cancers diagnosed in 2022, leaving the majority of cancers without available screening programs.^1^ In 2022, liver cancer was the third leading cause of cancer-related death globally (7.8% of total deaths) and ranked sixth in incidence rates (4.3% of all cancer sites).^1^ Pancreatic cancer has one of the worst prognoses and is responsible for nearly 5% of global cancer mortality, making it the sixth leading cause of cancer death.^1^ Age-standardized incidence rates (ASIRs) of several cancer types are increasing worldwide, such as liver cancer, kidney cancer, and non-Hodgkin lymphoma (NHL).^50^ Despite these increasing trends, screening programs do not exist for these cancer types. Reductions in mortality rates for bladder cancer and NHL have been observed and attributed to treatment improvements.^1^ Despite rising incidence, these cancers are often being diagnosed symptomatically at a later stage. The development of early detection tests combined with improved treatments could have notable population-level benefits.

The ability to detect multiple cancer types with a single test can mitigate the lack of existing screening programs for less common cancers. Recent studies evaluating the efficacy and accuracy of MCEDs reported detection of 5 to over 50 cancer types (Table 1).^14,51,52^ Evaluations of the Galleri^TM^ MCEDs report sensitivities of 80% or higher for cancers of the anus (81.8%, 18/22), ovary (83.1%, 54/65), pancreas (83.7%, 113/135), esophagus (85%, 85/100), head and neck (85.7%, 90/105), and liver and bile duct (93.5%, 43/46).^14^ Generally, sensitivity by stage for each cancer type varied and increased with advanced stages: anus (stage I: 25.0%, 1/4; stage II: 75.0%, 3/4; stage III: 100.0%, 13/13; stage IV: 100.0%, 1/1), ovary (stage I: 50.0%, 5/10; stage II: 80.0%, 4/5; stage III: 87.1%, 27/31; stage IV: 94.7%, 18/19), pancreas (stage I: 61.9%, 13/21; stage II: 60.0%, 12/20; stage III: 85.7%, 18/21; stage IV: 95.9%, 70/73), esophagus (stage I: 12.5%, 1/8; stage II: 64.7%, 11/17; stage III: 94.1%, 32/34; stage IV: 100.0%, 40/40), head and neck (stage I: 63.2%, 12/19; stage II: 82.4%, 14/17; stage III: 84.2%, 16/19; stage IV: 96.0%, 48/50), and liver and bile duct (stage I: 100.0%, 6/6; stage II: 70.0%, 7/10; stage III: 100.0%, 9/9; stage IV: 100.0%, 20/20).^14^ MCEDs, could broaden the number of screen-detectable cancers. Further, the Galleri^TM^ test has a high specificity of 99.5% (95% CI: 99.90-99.8), indicating a low false-positive rate of 0.5%, which may reduce the number of individuals exposed to additional unnecessary diagnostic procedures. This gives an advantage to MCEDs when compared to current single-cancer screening tests that typically prioritize sensitivity over specificity. A recent study used stage-specific incidence and survival data from the United States Surveillance, Epidemiology, and End Results (SEER) program to model the benefits and harms of MCED screening. Hubbell et al.’s findings state that MCEDs could intercept 312 (26%) to 485 (41%) cancer cases per year per 100,000 persons.^53^ In regard to modeled harms, Hubbell et al. report an estimated 692 false positive cases per year per 100,000 persons.^53^ More research is needed to evaluate the impact on false positives and overdiagnoses prior to MCED evaluation as a population-based test in the United States and Canada. The ability for MCEDs to detect many cancer types, while maintaining a low false-positive rate of 0.5%, may help to increase the overall population detection rate.

## Evidence Gaps and Potential Pitfalls

While early detection is generally recognized as beneficial and MCEDs may hold promise as a screening strategy, the evidence for MCEDs is limited. Much remains unknown about the clinical utility of these tests, such as the specific impacts of MCEDs on mortality and other clinical outcomes.^11^

Through our literature review, we have summarized potential obstacles to MCEDs future introduction with MCED testing into three notable pitfalls.

### Pitfall #1: Early detection does not always ensure a more favourable outcome

There is potential for MCEDs to increase anxiety or emotional stress, especially in the detection of cancers where no effective treatment exists or where early detection does not improve outcomes.^25^ For example, pancreatic cancers are generally diagnosed at advanced stages (III or IV), with 80%–90% of patients presenting with unresectable tumours at diagnosis.^54^ Largely due to the low incidence of the disease, current treatment options are limited, and survival outcomes are poor for pancreatic cancer, which may diminish the perceived benefits of early detection.^2^ However, with improved detection better treatments could be developed. Conversely in the absence of effective therapeutic options, an earlier diagnosis may be seen as an extended period of medical procedures, fear, and subsequent healthcare complications.^25^ The psychological toll on a patient’s life when they are diagnosed with cancer cannot be underestimated, and it is essential to weigh the risks and benefits before administering testing which may not find clinically actionable cancers. There are also scenarios where a detected cancer would not improve a person’s quality of life or risk of premature death, meaning that the utility of the detection is greatly diminished.^2^ Overdiagnosis of indolent cancers may also result in unnecessary psychological harms and overtreatment. Each cancer diagnosis and subsequent treatment impacts individuals and increases the strain on healthcare costs and resources, and so new screening modalities and programs must be carefully evaluated to ensure overdiagnosis is avoided.

### Pitfall #2: False positives and false negatives can impart a significant toll to healthcare spending and patient wellbeing

The advent of MCEDs elevates the concern for false-positive results that lead to unnecessary diagnostic tests and procedures, increased risk of adverse events, and unnecessary anxiety.^12^ False positives from MCEDs may also increase doubts in beliefs of the accuracy of MCEDs and discourage future participation in screening.^25^ In addition to actual false positives, cancers which are detected by MCEDs before conventional detection is possible may be falsely labelled as false positives, further casting doubt on MCEDs. MCEDs are useful for their ability to both detect a cancer and locate the TOO across many bodily systems.^9^ However, this raises an issue when false positives occur, because if cancer is not found in the predicted TOO, there is still a need to examine other organs to ensure that a cancer case is not being missed.^12^ This could potentially result in a time-intensive, invasive and expensive set of procedures to rule out cancer in other likely tissues of origin, which also may not have cancer. However, further investment into research and development of MCEDs could increase reliability and reduce the risk of false positives, partially solving these concerns. Additionally, in some cases an MCED could detect a cancer at an earlier stage than a conventional test. This could be falsely interpreted as a false positive, meaning that the cancer would not be identified and treated early. Research and planning would be required to determine the best course of action when specific early cancer tests have differing results to MCEDs.

Another potential issue with MCEDs is the prioritization of specificity over sensitivity in many of the studies examined (Table 1). While this can help to avoid false positives, it consequently increases the probability of false negatives. If MCED testing was used exclusively, this could lead to greater numbers of cancer cases going undetected.

### Pitfall #3: Patients may falsely believe that only an MCED test is needed, leading to a lack of adherence to existing screening protocols

Another potential issue with the introduction of MCEDs is overreliance on these new tests and subsequent reduction in conventional screening techniques. As previously discussed, MCEDs have the potential to achieve higher participation rates than conventional methods. However, overconfidence in these tests may lead patients to forgo cancer type-specific tests such as colonoscopies and mammograms, believing the MCED was sufficient. Individual screening tests still have benefits compared to MCEDs, including higher sensitivity in some cases, and the potential to reduce cancer risk (i.e. removing pre-cancerous polyps during a colonoscopy).^55^ Patients are likely to prefer the simpler procedure involved with MCEDs and seek to avoid existing more-invasive screening tests.^25^ This could result in undetected cancer cases in organs which have screening programs, negating some of the benefits of MCEDs. If MCEDs are introduced, healthcare providers will need to emphasize that they may be delivered in addition to traditional screening approaches, not as a substitute.

## Conclusions

MCEDs may help to address current gaps in cancer screening, including low participation rates, disparities among underserved populations, emerging trends in younger demographics, and the absence of screening tests for many cancer types. The uptake of MCEDs would enhance cancer prevention through improving and expanding current population-based screening approaches. By offering a comprehensive approach to detecting multiple cancer types with a single test, MCEDs have the potential to increase participation, improve access to screening for marginalized communities, and address the lack of screening options for less common cancers. While early results appear promising, there is currently limited evidence regarding the population-level performance of MCEDs. Further qualitative research is needed to determine patient attitudes toward MCED screening, as this will determine how effective these tests could be in real-world, population-based settings. Ultimately, larger implementation trials will be required to evaluate feasibility and logistics or MCEDs in health care systems to fully assess the potential of MCEDs in transforming cancer screening and prevention efforts.

## Appendix

### Abbreviations

ASIRs: Age-standardized incidence rates
cfDNA: Cell-free tumour DNA
EACS: Enhanced access to cervical and colorectal cancer screening
MCEDs: Multi-cancer early detection tests
NHL: Non-hodgkin lymphoma
PCPs: Primary care providers
SCEDs: Single-cancer early detection tests
SEER: Surveillance, epidemiology, and end results
TOO: Tissue of origin
USPSTF: U.S preventive services task force.
